# Additive Manufacturing of Silicon Nitride Ceramic Floatation Spheres with Excellent Mechanical Properties

**DOI:** 10.3390/ma12172717

**Published:** 2019-08-24

**Authors:** Hai Qi, Chenggui He, Peizhi Zhang, Weiyue Han, Fangquan Guo, Fen Wu, Miaofeng Du

**Affiliations:** Shanghai Key Laboratory of Engineering Materials Application and Evaluation, Shanghai Research Institute of Materials, Shanghai 200437, China

**Keywords:** silicon nitride, additive manufacturing, pressure housings, floatation spheres, full ocean depth, mechanical properties

## Abstract

Silicon nitride (Si_3_N_4_) ceramic materials are increasingly being used in deep-sea pressure-resistant applications because of their high compressive strength-to-weight ratio. In the present study, Si_3_N_4_ ceramic floatation spheres with an outer diameter of approximately 101 mm are successfully batch produced and evaluated. The implementation method was to prepare Si_3_N_4_ ceramic hemispherical housings and pair them together. In order to improve the safety of the joint, the hemispherical Si_3_N_4_ housings were gradually thickened from 1.80 to 2.50 mm at the equator near the joining surface, based on a 3D model with additive manufacturing technology. The weight-to-displacement ratio of the prepared floatation sphere is approximately 0.34 g/cm^3^. The flexural strength, compressive strength of the material and the collapse strength of a number of Si_3_N_4_ floatation spheres were tested to be 1150, 3847, and 205 MPa, respectively, to confirm the reliability of the process. Additional sustained and cyclic hydrostatic pressure tests simulating the full ocean depth working conditions are carried out on several Si_3_N_4_ floatation spheres, which perform very well and do not fail.

## 1. Introduction

Buoyancy modules and housings utilized in underwater vehicles demand deep-sea pressure-resistant materials with high compressive strengths and low densities to meet the requirements of water pressure resistance and reduce equipment weight. Ceramic materials have a great advantage as deep-sea pressure-resistant materials because of their high compressive strength-to-weight ratio relative to high-strength steels and titanium alloys. Alumina (Al_2_O_3_) ceramics were first used to produce hollow spheres as buoyancy modules [1,2,3] and external pressure housings for deep-sea vehicles [4]. However, compared to silicon nitride (Si_3_N_4_) ceramics (see in Table 1), Al_2_O_3_ ceramics are not the best choice in terms of high compressive strength and low density. Si_3_N_4_ ceramics have a high compressive strength-to-weight ratio and are, therefore, favored by many researchers. Takagawa [5] compared several structural ceramics, confirmed Si_3_N_4_ ceramics as the best material for autonomous underwater vehicle (AUV) pressure-resistant housing, and prepared a scale model for a pressure test of 118 MPa. In some studies [6,7,8], Si_3_N_4_ ceramics were used as pressure housings for ocean bottom seismometers, and a series of pressure tests were conducted to confirm their reliability.

In the production of Al_2_O_3_ ceramic floatation spheres [1,2,3], seamless hollow spheres were prepared by rotation casting and pressureless sintering after drying. However, for Si_3_N_4_ ceramics, the seamless hollow sphere greenbody is not available. It would be damaged in the high-temperature and high-pressure conditions during gas pressure sintering, which is necessary to apply to acquire high strength Si_3_N_4_ materials [9]. Consequently, fabricating two hemispherical housings and joining them together is the best way to prepare Si_3_N_4_ ceramic floatation spheres at present. However, the presence of joints causes the ceramic spheres to fail at a lower pressure than it would in the absence of joints [10]. As explored by Yano and Takagawa [11], a tiny deformation of the circular joining surface might seriously deteriorate the collapse strength because of the small overlapping area at the joining part. Due to the poor machinability of the ceramic materials, it is very difficult to precisely control the pairing of the two hemispherical Si_3_N_4_ housings through machining, which will result in extremely high costs, especially for batch production. Nevertheless, with the development of preparation processes for ceramics, a novel technology—additive manufacturing (AM, also known as 3D printing)—makes batch production of the Si_3_N_4_ floatation spheres feasible.

The ISO/ASTM 52900:2015 standard of AM technologies introduces AM as the process of joining materials layer upon layer based on a 3D model. Theoretically, AM can build a part from any 3D model without following traditional manufacturing methods. It not only has the advantage of reducing manufacturing costs but also gives designers greater freedom. Breddermann et al. [12] investigated the possibility of building pressure housings in the AM process, and they demonstrated that the housings could be produced even though the reliability still needed to be confirmed.

**Figure 1:**
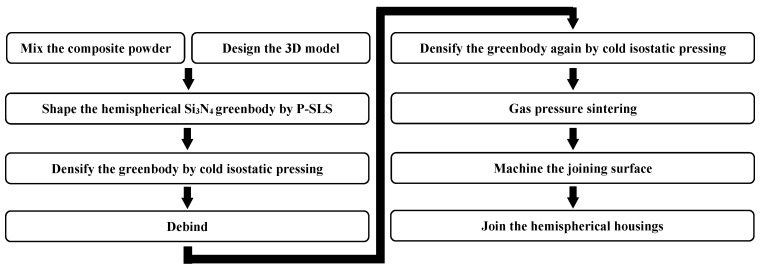
Preparation process of Si_3_N_4_ ceramic floatation spheres.

The aim of this paper is to prepare Si_3_N_4_ ceramic floatation spheres with the aid of AM technology, therefore reducing cost and making batch production more feasible. As mentioned above, the implementation method is to prepare Si_3_N_4_ ceramic hemispherical housings first and then pair them together. In this study, the adopted process, which was explored by Deckers et al. [13], Liu et al. [14], and Liu et al. [15] with Al_2_O_3_ materials, is powder-based selective laser sintering (P-SLS, which belongs to indirect AM technologies as reviewed by Zocca et al. [16]) combined with cold isostatic pressing. The specific processing flow chart of preparing Si_3_N_4_ ceramic floatation spheres [17] is schematically shown in Figure 1. In addition, the flexural strength, compressive strength of the Si_3_N_4_ ceramic material, and collapse strength of a number of Si_3_N_4_ ceramic floatation spheres are tested to confirm the reliability of the process.

## 2. Materials and Methods

### 2.1. Composite Powder Preparation

The raw material was spray granulated [18] Si_3_N_4_ ceramic powder (provided by UBE Co., Tokyo, Japan, particle size: 50–100 μm) and was mechanically mixed with 20 wt% of epoxy resin powder (provided by Guangzhou Shinshi Metallurgy and Chemical Company LTD., Guangzhou, China, SH-E50) as a binder phase in a V-type mixer for printing.

### 2.2. Description of the 3D Model

**Figure 2:**
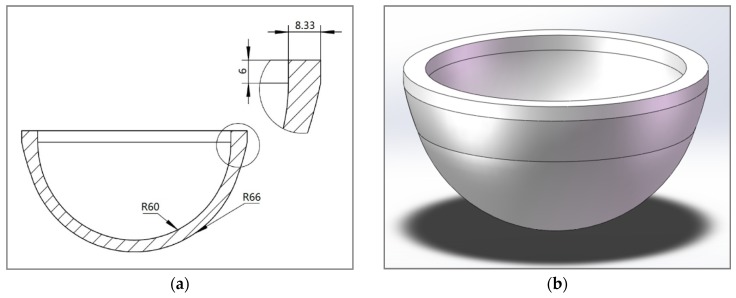
Technical sketch of the hemispherical greenbody. (**a**) CAD drawing; (**b**) 3D model.

The technical sketch of the 3D model is shown in Figure 2. After shrinking by cold isostatic pressing and sintering, the wall thickness of the hemispherical housing was expected to be 1.8 mm, and the region near the joining surface was expected to gradually thicken to 2.5 mm, which was determined by the maximum roundness error of the circular joining surface (about 0.7 mm). The purpose of thickening the hemispherical housing at the equator region was to reduce the impact of deformation of the circular joining surface and to improve the safety factor of the joint.

### 2.3. Powder-Based Selective Laser Sintering

The hemispherical housing greenbodies were printed using a HRPS-IV SLS system (made by Huazhong University of Science and Technology, Wuhan, China) equipped with a 55 W CO_2_ laser with a wavelength of 10.6 μm and a laser beam diameter of 200 μm. The laser power, scan speed, scan spacing, and layer thickness were 7.7 W, 2500 mm/s, 200 μm, and 100 μm, respectively.

### 2.4. Cold Isostatic Pressing and Debinding

The hemispherical housing greenbodies were cold isostatic pressed for 2 min at 260 MPa and then debinded for 2 h at 500 °C. After debinding, cold isostatic pressing was conducted again on the greenbodies.

### 2.5. Sintering, Machining, and Joining

The debinded and cold isostatic pressed hemispherical housing greenbodies were sintered for 1 h at 1750 °C in 5 MPa of N_2_. Then, the joint surface of the sintered hemispherical housing was machined by grinding and polished with diamond paste of 0.5 μm. The joining of the hemispherical housings was carried out at a temperature of 1550°C for 1 h in 0.1 MPa of N_2_.

### 2.6. Measurements and Test Procedure

The wall thickness of the hemispherical housings was measured by a micrometer (made by Shanghai Tool Works Co., LTD., Shanghai, China). The density of the resulting ceramic materials and the weight-to-displacement ratio of the floatation spheres were determined through the Archimedes method by an electronic balance (MP21001, made by Shanghai Sunny Hengping Scientific Instrument Co., LTD., Shanghai, China). The microstructure of the hemispherical housings in different processes was observed by scanning electron microscopy (SEM, VEGA 3 SBU, made by TESCAN, Brno, Czech). The joining status of the hollow spheres was observed by industrial computed tomography (CT, V|tome|x, made by GE, Boston, MA, USA).

The flexural strength (three-point) *σ_F_* of the Si_3_N_4_ material was tested on sixteen 3 × 4 × 38 mm^3^ samples (surface finished by grinding and polishing) at room temperature to evaluate the quality of the material. The calculation formula is

(1) $$\sigma_{F}=\frac{3FL}{2bd^{2}}$$

where *F* is the fracture load, *L* is the fixture outer span (30 mm), *b* is the specimen width, and *d* is the specimen thickness. The Weibull modulus *m* was also calculated.

The compressive strength *σ_C_* of the Si_3_N_4_ material was tested on ten 5 × 5 × 12.5 mm^3^ samples (surface finished by grinding and polishing) at room temperature to provide data to predict the collapse strength of the floatation spheres. The calculation formula is

(2) $$\sigma_{C}=\frac{P}{A}$$

where *P* is the compressive breaking load, and *A* is the specimen sectional area. 

The collapse strength of the Si_3_N_4_ ceramic floatation spheres was tested in a 500 MPa rated pressure tank (LDJ180-250-500, made by MUCH Machinery Co., LTD., Foshan, China). The pressure was read with a type PTB702 pressure sensor (accuracy 1.2 MPa, made by Guangzhou Compass Sensor Instrument Co., LTD., Guangzhou, China). In addition, sustained and cyclic hydrostatic pressure tests were performed.

## 3. Results and Discussion

### 3.1. Preparation

**Figure 3:**
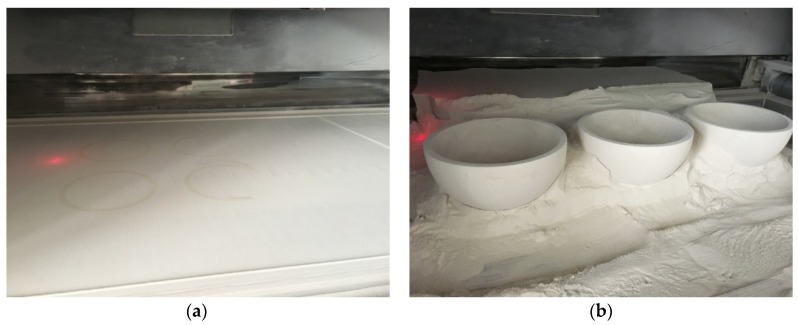
(**a**) Printing the hemispherical greenbodies in the additive manufacturing (AM) equipment, and (**b**) the printed hemispherical greenbodies.

Six hemispherical housings as a batch were printed by the SLS system based on the 3D model, as shown in Figure 3a. During printing, the epoxy resin powder was selectively melted by a laser light to bond the Si_3_N_4_ ceramic particles together to form parts. After removing the non-sintered composite powder, which could be reused, the greenbodies were taken out from the powder bed, as shown in Figure 3b.

**Figure 4:**
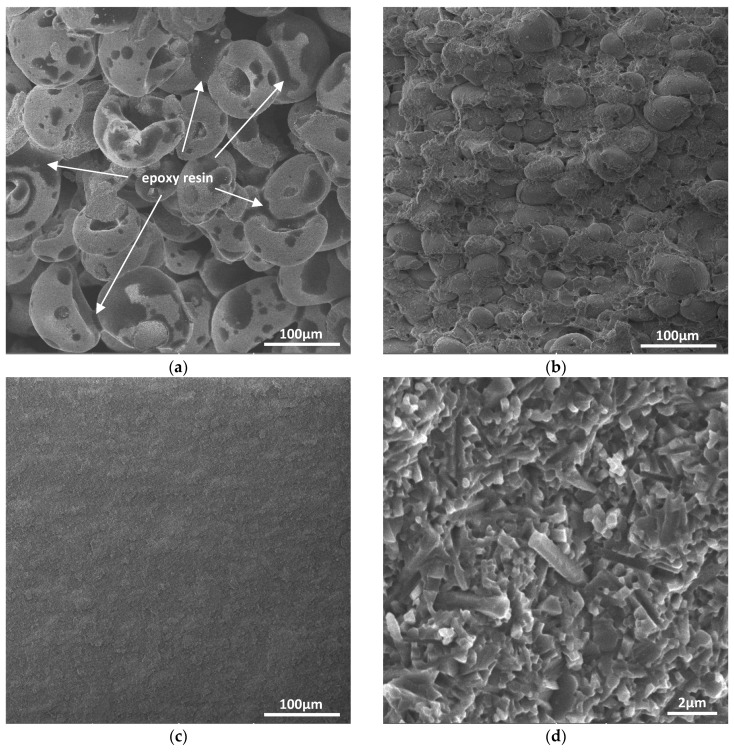
SEM micrographs of the hemispherical housings in different processes. (**a**) The printed greenbody before cold isostatic pressing CIP, (**b**) the first CIPed and debinded greenbody, (**c**) the second CIPed greenbody, and (**d**) the sintered Si_3_N_4_ body.

Figure 4a shows that the Si_3_N_4_ particles (spray granulated) adhered to each other with epoxy resin after printing. The loose structure of the printed greenbody was also observed. Since the density and uniformity of the greenbodies shaped by P-SLS were too low to obtain a high quality sintered body, cold isostatic pressing was applied to improve the strength and reliability of the material. Then, the greenbodies were debinded to remove the epoxy resin. Figure 4b shows the microstructure of the first cold isostatic pressed and debinded greenbody. Notably, lots of pores were generated in removing the high-content binder. In order to eliminate the pores, cold isostatic pressing was applied again to further improve the density and strength of the greenbodies. After the second cold isostatic pressing, the greenbody was firmer and much more uniform (see Figure 4c).

**Figure 5:**
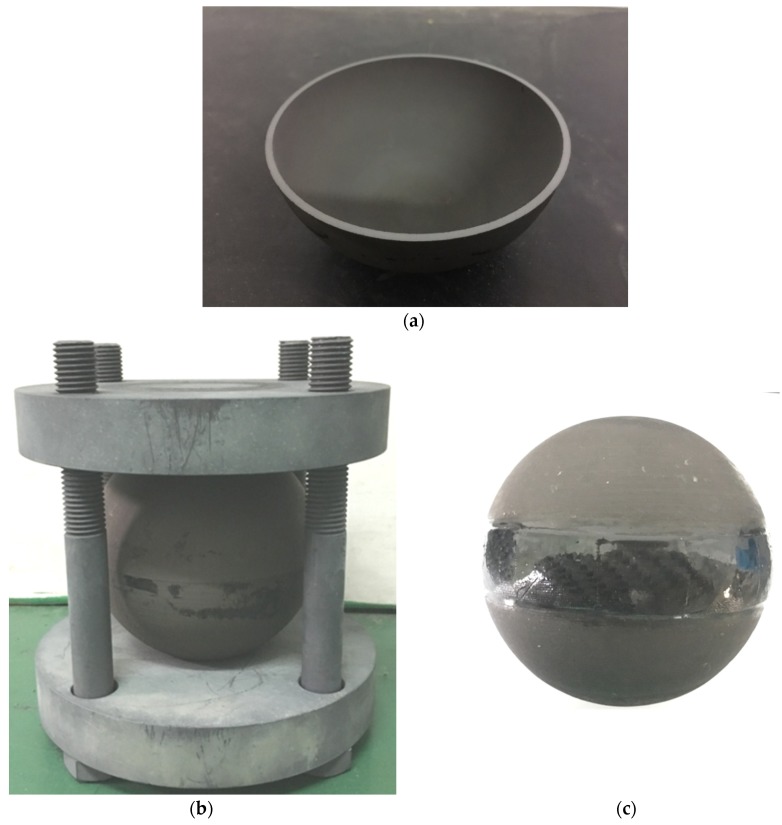
(**a**) A sintered and machined Si_3_N_4_ housing, (**b**) the graphite fixture for joining, and (**c**) a floatation sphere coated with the carbon fiber material.

After cold isostatic pressing and debinding, the greenbodies were gas pressure sintered. Figure 4d shows the micrograph of the sintered Si_3_N_4_ body. The elongated rod-like grains provide toughening mechanisms that make Si_3_N_4_ materials have better mechanical properties than Al_2_O_3_. The density of the sintered Si_3_N_4_materials was measured to be 3.23 g/cm^3^, and the porosity was close to 0% according to the theoretical density of the material, which means the ceramic materials had no pore defects. Next, the sintered hemispherical housings were machined and polished to prepare for joining together, as shown in Figure 5a. According to quite a few bonding phenomena, which were often observed between samples in contact with each other in some heat treatment experiments of Si_3_N_4_ ceramics, the hemispherical housings were joined at a high temperature (1550 °C) with a graphite fixture, as shown in Figure 5b. The advantage of heat joining is that it is much more durable than joining with organic adhesives. Heat treatment can also eliminate the stress concentration on the joining surface. In addition, in order to avoid water penetrating into the seam of the joined housings in deep-sea, high-pressure conditions, the seam was coated with carbon fiber material to seal and protect the interface, as shown in Figure 5c.

This study prepared seventeen Si_3_N_4_ ceramic floatation spheres for pressure tests, sixteen samples for testing the flexural strength of the Si_3_N_4_ material, and ten samples for testing the compressive strength of the Si_3_N_4_ material.

### 3.2. Evaluation

**Figure 6:**
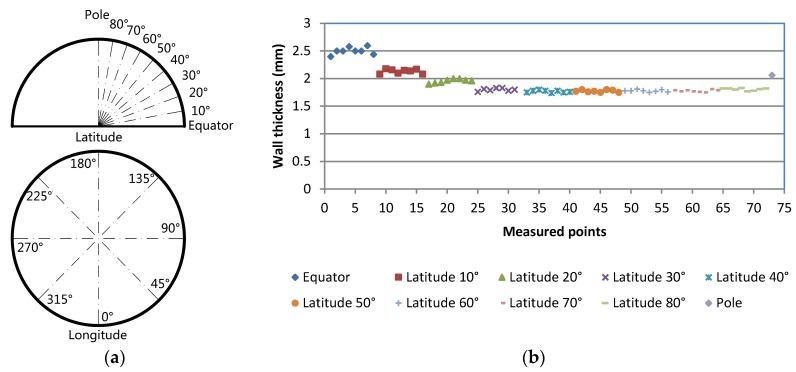
Wall thickness measurement results of a sintered and machined hemispherical housing. (**a**) Schematic diagram of wall thickness measured points of the hemispherical housing. (**b**) Measurement results.

The wall thickness distribution of the hemispherical housing after sintering and machining is shown in Figure 6. The wall thickness of the measured hemispherical housing ranged from 1.77 ± 0.02 mm to 2.50 ± 0.06 mm, and the thickened region ranged from 1.80 ± 0.02 mm to 2.50 ± 0.06 mm. Wall thickness measurements were basically consistent with the expected value, except for the pole location (2.06 mm), which might be attributed to the density inhomogeneity of the greenbody caused by the AM process. The diameter of the uncoated floatation spheres and the weight-to-displacement ratio of the coated floatation spheres were also measured, as shown in Table 2 and Table 3. 

**Figure 7:**
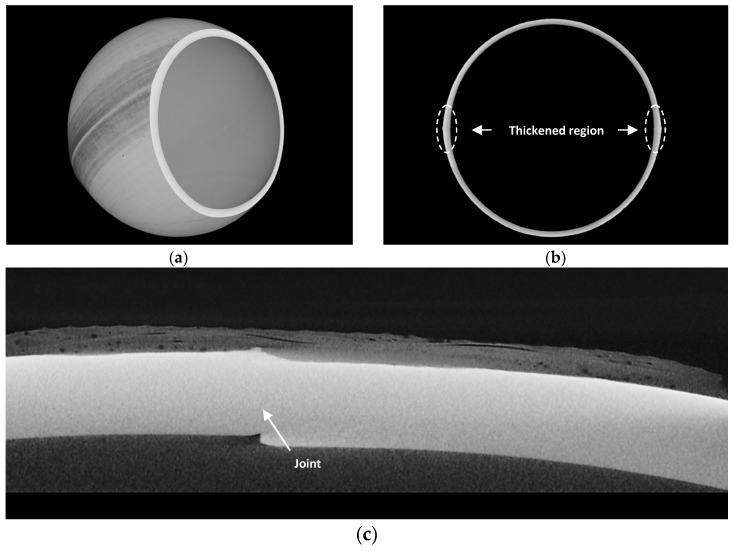
Industrial computed tomography (CT) images of a Si_3_N_4_ floatation sphere. (**a**,**b**) CT scanning. (**c**) Slight misalignment at the joint.

A joined floatation sphere was scanned by industrial CT. No pores or other defects were observed in CT scanning (resolution 100 μm). CT images show that the two hemispherical housings were joined well (Figure 7b), but slight misalignment between the two housings can be observed at the joint in Figure 7c, which should be the result of the roundness error of the circular joining surface.

**Figure 8:**
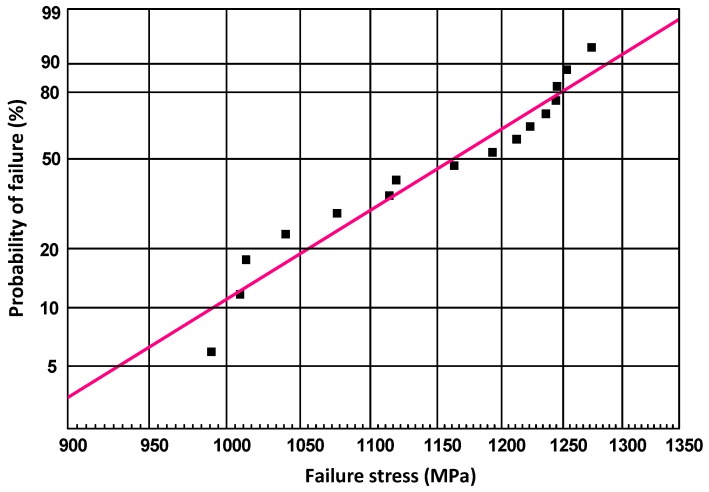
Weibull plot for flexural strength of the Si_3_N_4_ material fabricated based on powder-based selective laser sintering (P-SLS).

The flexural strength of the Si_3_N_4_ material was tested using sixteen samples (3 × 4 × 38 mm^3^, prepared with the same process as the hemispherical housings) at room temperature. The result is shown in Figure 8. The average value of the flexural strength was 1150 ± 96 MPa, which means the Si_3_N_4_ material fabricated in this study achieved the same level as that of traditional processes (see in Table 1). The Weibull modulus *m* was calculated to be 11.8, which is close to the commercial material (Level I) in ASTM F2094. Therefore, the reliability of the material prepared by the process was confirmed.

The compressive strength of the Si_3_N_4_ material was tested using ten samples (5 × 5 × 12.5 mm^3^, prepared with the same process as the hemispherical housings) at room temperature. The average value of the compressive strength was 3847 ± 249 MPa (ranged from 3467 MPa to 4239 MPa), which was also close to the Si_3_N_4_ material prepared by traditional processes (see in Table 1). As the compressive strength was tested, the collapse strength *P_C_* of an ideal hollow sphere could be estimated by the formula [3,12]

(3) $$P_{C}=\frac{2\sigma_{C}t}{r}$$

where *σ_C_* is the compressive strength of the material, *t* is the wall thickness of the ceramic sphere, and *r* is the spherical radius. For the Si_3_N_4_ floatation spheres prepared in this study, *t* is 1.77 mm (the minimum value), *r* was 50.79 mm, and the calculated theoretical collapse strength of the floatation sphere was 268 MPa.

### 3.3. Pressure Tests

A total of seventeen Si_3_N_4_ floatation spheres prepared in this study were tested. Ten of them were tested to collapse, and the others were tested with sustained and cyclic hydrostatic pressure. The collapse strength ranged from 164 MPa to 236 MPa, and the average value was 205 ± 24 MPa, as shown in Table 2. The results of the sustained and cyclic hydrostatic pressure tests are shown in Table 3. The test conditions were much closer to the actual working conditions, and all seven Si_3_N_4_ floatation spheres performed very well and did not fail.

The measured collapse strength was much lower than the theoretical value calculated above (268 MPa), which means there is still room to improve the shape’s accuracy. For example, the sphericity of the hemispherical housings, which was not analyzed in detail in this study, has a large impact on collapse strength [19]. To improve the collapse strength and reduce data deviation, more details, such as sphericity, machining precision, and so on, in the process will be investigated and controlled in our future work.

For the Si_3_N_4_ floatation spheres prepared in this study, the safety factors of all the tested ten Si_3_N_4_ floatation spheres were high enough (from 1.5 to 2.2) for the full ocean depth conditions (maximum water pressure is 110 MPa). Therefore, we think this process of preparing Si_3_N_4_ floatation spheres was confirmed to be reliable for practical full ocean depth applications. Because of the high strength of the material, the performance of the Si_3_N_4_ floatation spheres was better than the seamless Al_2_O_3_ floatation spheres tested by Weston et al. [2]. Furthermore, based on the results in this study, it is clear that Si_3_N_4_ ceramic materials, with their high strength and low density, show great potential to serve in deep-sea pressure-resistant applications.

Since the reliability of the process adopted in this study was confirmed by testing the flexural strength, compressive strength of the material, and the collapse strength of the floatation spheres, it promises to develop the process in other deep-sea pressure-resistant applications and give ocean engineers greater freedom in designing pressure housings. It is worth noting that the process is not limited to Si_3_N_4_ ceramic materials. Other structural ceramic materials, such as Al_2_O_3_, silicon carbide (SiC), zirconia (ZrO_2_), zirconia toughened alumina (ZTA), and so on, are also available to prepare pressure housings for various requirements and working conditions.

## 4. Conclusions

This work demonstrates a new and feasible approach to realize batch production of deep-sea pressure-resistant floatation spheres with Si_3_N_4_ ceramic material. Based on AM technology, gradually thickened hemispherical Si_3_N_4_ housings (from 1.77 ± 0.02 mm to 2.50 ± 0.06 mm) were successfully prepared. After the joining surfaces were machined, the hemispherical housings were paired and joined to fabricate floatation spheres. In addition, the flexural strength (1150 ± 96 MPa) and the compressive strength (3847 ± 249 MPa) tested on samples prepared with the same process as the hemispherical housings confirmed the quality of the material. A series of pressure tests on a number of Si_3_N_4_ ceramic floatation spheres (the weight-to-displacement ratio was 0.34 ± 0.01 g/cm^3^) were conducted. Although the collapse strength (205 ± 24 MPa) of the floatation spheres was lower than the calculated theoretical value (268 MPa, according to the compressive strength of the material), it was high enough to meet the full ocean depth conditions. In the sustained and cyclic hydrostatic pressure tests, all tested floatation spheres performed well and did not fail.

## Figures and Tables

**Table 1:** Comparison of material properties between Si_3_N_4_and Al_2_O_3_ (commercial reference values).

Properties	Si_3_N_4_ (Gas Pressure Sintered)	Al_2_O_3_ (High Purity, ≥99%)
Density (g/cm^3^)	3.2	3.9
Flexural Strength (3 point, MPa)	900–1200	300–500
Compressive Strength (MPa)	3000–4000	2000–3500
Fracture Toughness (MPa·m^1/2^)	6.5	4.0
Young’s Modulus (GPa)	300	380
Poisson’s Ratio	0.27	0.23

**Table 2:** Diameter, weight-to-displacement ratio, and collapse strength of the Si_3_N_4_ floatation spheres.

Sample Number	Diameter (Uncoated, mm)	Weight-to-Displacement Ratio (Coated, g/cm^3^)	Pressure (MPa)	Remarks
1	101.43	0.35	200	Collapsed
2	101.52	0.35	236	Collapsed
3	101.72	0.34	216	Collapsed
4	101.66	0.35	186	Collapsed
5	101.62	0.34	198	Collapsed
6	101.56	0.34	164	Collapsed
7	101.51	0.34	218	Collapsed
8	101.62	0.33	173	Collapsed
9	101.78	0.33	234	Collapsed
10	101.33	0.35	225	Collapsed
Average value	101.58	0.34	205	-

**Table 3:** Results of sustained and cyclic hydrostatic pressure tests.

Sample Number	Diameter (Uncoated, mm)	Weight-to-Displacement Ratio (Coated, g/cm^3^)	Test Condition	Remarks
11	101.55	0.34	Sustained ^1^	Not failed
12	101.31	0.35	Sustained	Not failed
13	101.79	0.33	Sustained	Not failed
14	101.83	0.33	Sustained	Not failed
15	101.42	0.35	Sustained	Not failed
16	101.63	0.33	Cyclic ^2^	Not failed
17	101.59	0.34	Cyclic	Not failed

^1^ 145 MPa held for 10 h; ^2^ 115 MPa held for 1 min and cycled 1000 times.
